# Impact of anatomic complexity of congenital heart disease on growth retardation: a large retrospective cohort study

**DOI:** 10.3389/fcvm.2026.1907307

**Published:** 2026-09-10

**Authors:** Xi Yang, Siyu Liang, Xin Tang, Yutong Liu, Linyao Wang, Shi Chen, Hanze Du, Zengren Zhao, Hui Pan, Huijuan Ma

**Affiliations:** 1 Department of Endocrinology, The First Hospital of Hebei Medical University, Shijiazhuang, Hebei, China; 2 Department of Endocrinology, Baoding First Central Hospital, Baoding, Hebei, China; 3 Department of Endocrinology, Key Laboratory of Endocrinology of National Health Commission, Translation Medicine Center, Peking Union Medical College Hospital, Peking Union Medical College, Chinese Academy of Medical Sciences (PUMCH, PUMC & CAMS), Beijing, China; 4 Eight-year Program of Clinical Medicine, PUMCH, PUMC & CAMS, Beijing, China; 5 Gastrointestinal Disease Centre, Hebei Key Laboratory of Colorectal Cancer Precision Diagnosis and Treatment, The First Hospital of Hebei Medical University, Shijiazhuang, Hebei, China

**Keywords:** anatomic complexity, congenital heart disease, growth retardation, short stature, underweight

## Abstract

**Background:**

Growth retardation (GR) is a significant comorbidity in children with congenital heart disease (CHD), particularly those with complex CHD. This study aimed to characterize the growth patterns of pediatric CHD patients and to identify the factors associated with GR.

**Methods:**

This retrospective cohort study included CHD patients aged ≤18 years from the First Hospital of Hebei Medical University between January 2014 and March 2024. Patients were classified into complex or non-complex CHD groups based on echocardiographic diagnoses. Clinical, laboratory, and echocardiographic parameters were compared. Independent factors associated with GR were identified and used to construct a nomogram for identifying the likelihood of existing GR.

**Results:**

A total of 2,784 patients were included (260 with complex CHD and 2,524 with non-complex CHD). Patients with complex CHD had higher prevalence of growth impairment than those with non-complex CHD. GR (35.4% vs. 16.0%), short stature (30.0% vs. 15.1%), and underweight (27.3% vs. 13.4%) were more common in complex CHD than in non-complex CHD patients. Complex CHD, younger age, presence of pulmonary arterial hypertension, and hypoalbuminemia were identified as independent factors associated with GR. A nomogram incorporating these factors demonstrated performance in screening for prevalent GR, with an area under the receiver operating characteristic curve of 0.75 in the temporal validation cohort.

**Conclusions:**

The prevalence of GR is high in children with CHD, especially among those with complex CHD. Our findings highlight the necessity of integrating routine growth surveillance into the standard of care for this population. The nomogram facilitates the identification of children with a high probability of GR, enabling timely intervention.

## 1 Introduction

Congenital heart disease (CHD) is a congenital malformation caused by abnormal embryonic development of the heart and great vessels, accounting for 25% of all congenital malformations (1). In China, the reported detection rate of CHD is estimated to range from 2.4% to 10.4% (2). Significant advancements in cardiac surgery and interventional therapies have dramatically improved prognoses of CHD, with survival rates now exceeding 90% (3–5). Consequently, the clinical and research focus has necessarily shifted towards managing CHD-related comorbidities and complications.

Among them, growth retardation (GR) has gradually attracted attention as a clinical concern affecting the quality of life of CHD patients. Beyond impairing physical development, GR is associated with significant psychosocial and functional deficits. Previous studies have demonstrated that, compared with their normal-height peers, children and adolescents with GR face more severe psychosocial challenges and exhibit poorer social adaptation (6). Moreover, this adverse impact persists into adulthood, with short-statured individuals continuing to report significantly lower quality of life scores driven by ongoing psychosocial difficulties. Previous studies have found that compared with healthy children, patients with CHD exhibit a higher prevalence of GR (7), which frequently manifests as low weight and short stature. Indeed, some cohorts report that as many as 55.9% of children with CHD present with significant short stature (8–10).

The etiology of GR in CHD is multifactorial, including congenital factors such as genetic syndromes and intrauterine growth restriction, as well as acquired factors such as malnutrition, heart failure, chronic inflammatory state, and endocrine disorders (11–13). The prevalence and severity of GR vary among different types of CHD. Moreover, some studies believe that insufficient nutritional intake is the main influencing factor (14), and other studies believe that hypoxia is the factor associated with GR in CHD patients (15). Our previous studies support focusing on the effect of cardiac structural abnormalities on GR. We found that patients with complex CHD have the highest prevalence of GR, with a rate of 54.2% (16). However, a comprehensive scoring system to reliably assess the probability of GR in CHD is currently lacking.

In patients with CHD, GR is often an indicator of uncontrolled underlying chronic disease. Therefore, timely identification and correction of reversible causes are essential not only for achieving satisfactory final height but also for better adaption to society and improving the quality of life (17, 18).

We conducted a large-scale retrospective cohort study to: (1) identify factors associated with GR in patients with CHD, with a specific focus on the growth and development characteristics of patients with complex CHD; (2) develop and validate a model for identifying the likelihood of existing GR. We aimed to provide clinicians with a practical tool for the early detection, and stratification of children with CHD currently presenting with high-risk features for GR.

## 2 Material and methods

### 2.1 Study design

This single-center retrospective cohort study included patients with a diagnosis of CHD admitted to the First Hospital of Hebei Medical University, Hebei, China between January 2014 and March 2024. Data were extracted from the electronic medical record (EMR). Informed consent was obtained from all parents or legal guardians of the participants prior to their participation. The study protocol was approved by the Ethics Committee of the First Hospital of Hebei Medical University (approval number: [2024] No. 200, approval date: December 31, 2024).

### 2.2 Participants

Eligible participants were aged 0–18 years with a confirmed diagnosis of CHD. Since post-intervention hemodynamic improvements often trigger catch-up growth, which may confound the assessment of the impact of CHD on growth, this study focused on the pre-intervention growth status of patients with CHD. Patients were excluded if they met any of the following criteria: (1) incomplete medical records, defined as missing echocardiographic data or growth parameters (i.e., height or weight); (2) previous surgical or catheter intervention for their CHD; or (3) presence of significant comorbidities known to independently affect growth, such as tumor, liver failure, or renal failure.

The patient cohort was divided for model development and validation. A training cohort, comprising patients admitted from January 2014 to March 2021, was used to identify factors associated with GR and construct the nomogram. The validation cohort, including patients admitted from April 2021 to March 2024, was used for temporal internal validation of the model's performance in identifying prevalent GR.

### 2.3 Data collection and variables

All clinical and laboratory data were extracted from the EMR. The extracted variables included demographic and anthropometric data (gender, age, height, weight); laboratory test results [total protein (TP), albumin (ALB), total cholesterol (TC), low-density lipoprotein (LDL), carbon dioxide combining power, red blood cell count (RBC), hemoglobin (HGB), and prothrombin time); echocardiographic findings [pulmonary arterial hypertension (PAH), ventricular septal defect (VSD) size] and clinical outcomes, such as length of stay (LOS). Additionally, data on clinical presentation (e.g., cyanosis, wheezing) and pneumonia were collected. It is important to note that data on clinical presentation were systematically collected only for patients admitted prior to July 2018 (*n* = 1670). Therefore, any statistical analysis involving these specific variables was restricted to this sub-cohort. All other variables were analyzed across the entire study period.

Body mass index (BMI) was calculated as weight (kg) divided by height (m^2^). To normalize for age and sex, height and weight standard deviation scores (SDS) were calculated using the formula: SDS = (observed value − median reference value for age and sex)/standard deviation of the reference value. The reference data for these calculations were sourced from contemporary Chinese growth standards. For children under 7 years of age, the average height and weight corresponding to age and sex were referred to the Growth Standards for Children under 7 years of age issued by the National Health Commission of China in 2022 (19). For children 7 years and older, data from the Chinese National Survey released in 2009 were referenced (20).

According to clinical presentation and echocardiographic findings, all patients were divided into complex CHD and non-complex CHD. The complex CHD was defined according to established criteria (21), including cyanotic CHD, double-outlet ventricle, CHD requiring Fontan procedure, interrupted aortic arch, pulmonary atresia (all forms, including tetralogy of Fallot with pulmonary atresia), single-ventricle anatomy (including double-inlet left ventricle, tricuspid/mitral atresia, hypoplastic left heart, any other cardiac anatomic abnormality with a functionally single ventricle), congenitally corrected TGA (CCTGA, levo-TGA), dextro-TGA after atrial switch (Mustard/Senning) operation, dextro-TGA after Rastelli operation, truncus arteriosus, unrepaired or partially palliated cyanotic congenital heart defect. The non-complex CHD included atrial septal defect, ventricular septal defect, patent ductus arteriosus, coarctation of aorta, and mild, isolated pulmonary artery stenosis or dilatation.

To elucidate the age-stratified cross-sectional growth profiles of children with CHD, we analyzed anthropometric measurements across distinct age groups. These visualizations depicted height-for-age and weight-for-age, along with their corresponding SDS, and were further stratified by the specific subtype of CHD. Acknowledging the period from birth to 12 years as a critical window for physical development, our analysis was specifically focused on characterizing the growth patterns within this crucial age range.

### 2.4 Outcomes

The primary outcome of this study was the presence of GR at the time of hospital admission. The GR was defined as height or weight less than 2 SDS (−2 SDS) below the mean of a healthy population of the same race, age, and sex in a similar setting. The secondary outcomes were the presence of short stature (height-for-age SDS < −2) and underweight (weight-for-age SDS < −2).

### 2.5 Statistical analysis

All statistical analyses were performed using SPSS (Version 27.0, IBM, Armonk, New York, United State of America) and R software (Version 3.6.2, R Foundation for Statistical Computing, Vienna, Austria). Continuous variables were expressed as mean ± SDS or median (IQR) and compared using Student's *t*-test or the Mann–Whitney *U* test, depending on their distribution. Categorical variables were reported as frequencies and percentages and compared using Pearson's *χ*^2^ test or Fisher's exact test.

To identify associated factors of GR in CHD, a multivariable logistic regression model was constructed. First, a univariate logistic regression was performed for all candidate variables. Variables with a *P*-value <0.05 in the univariate analysis were selected for inclusion in a stepwise multivariable logistic regression model. Multicollinearity among variables was assessed using the variance inflation factor (VIF), with a threshold of VIF > 5 indicating significant collinearity. A nomogram was established based on the final selected variables. The receiver operating characteristic (ROC) curve and the area under the curve (AUC) were used to evaluate the performance of the nomogram. The consistency between the model-estimated probability and the observed probability was evaluated by a calibration plot. Decision curve analysis (DCA) was conducted to quantify the clinical utility and net benefit of the nomogram. *P* < 0.05 was considered statistically significant.

## 3 Results

### 3.1 Baseline characteristics and clinical outcomes of participants

**Figure 1:**
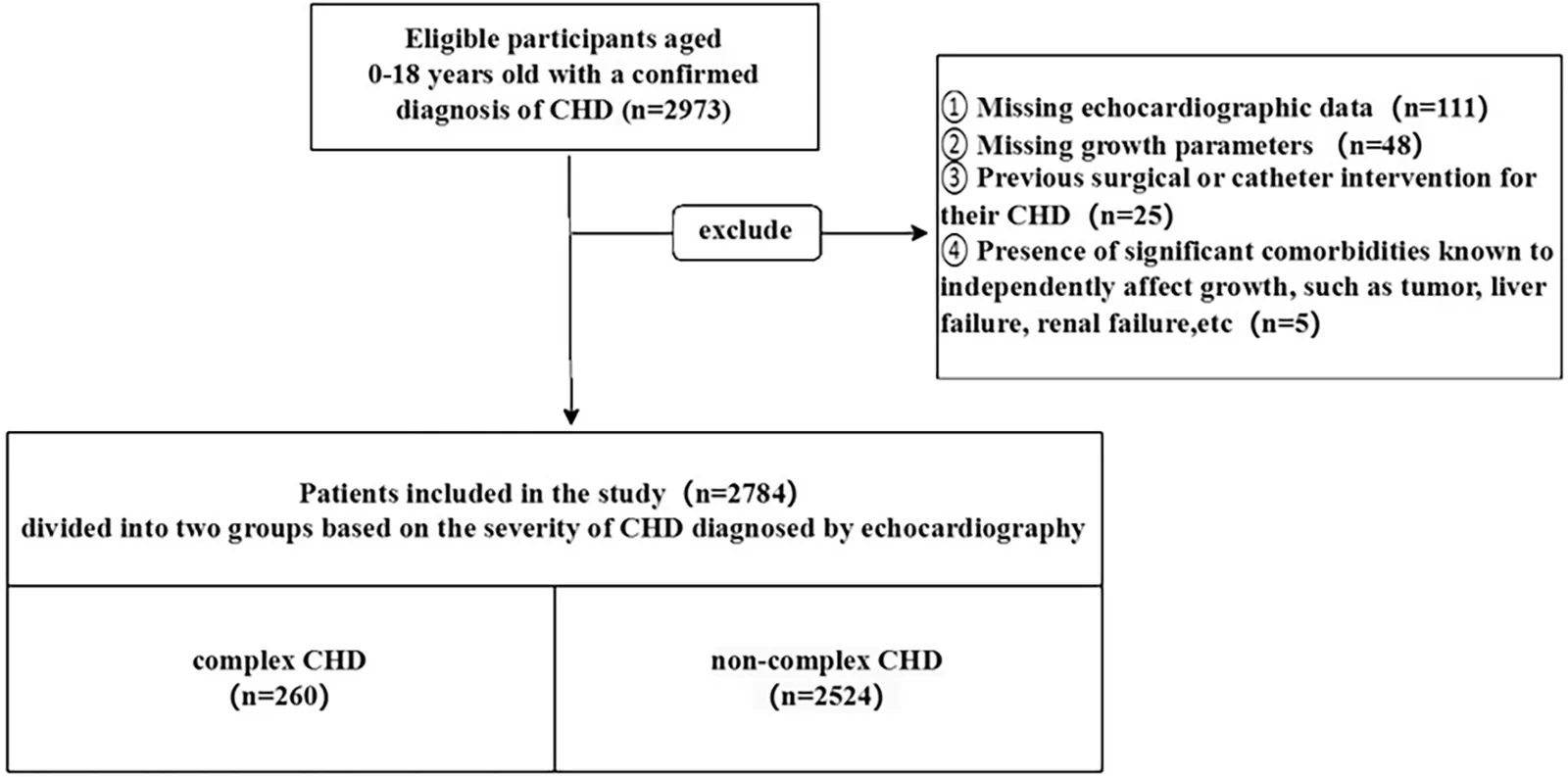
The flowchart of patients included in this study.

A total of 2,784 patients with CHD were included in the final analysis, comprising 260 patients with complex CHD and 2,524 patients with non-complex CHD (Figure 1).

Patients with complex CHD had higher prevalences of growth impairment than those with non-complex CHD. GR (35.4% vs. 16.0%), short stature (30.0% vs. 15.1%), and underweight (27.3% vs. 13.4%) were more common in complex CHD patients than in non-complex CHD patients. Clinically, patients with complex CHD presented more frequently with cyanosis (74.1% vs. 2.1%), wheezing (3.3% vs. 0.5%), and pneumonia (6.9% vs. 3.3%) (data available for *n* = 1,670 patients). The median VSD size was significantly larger in the complex group (11.9 vs. 2.1 mm). Laboratory analyses revealed that the complex CHD group had significantly poorer nutrition status, characterized by lower levels of TP, ALB, TC, and LDL. Conversely, this complex CHD group exhibited higher RBC and HGB, consistent with a state of chronic hypoxia (Table 1). Regarding clinical outcomes, patients with complex CHD had a longer median duration of hospitalization (18.00 vs. 13.00 days). In-hospital mortality was slightly higher in the complex CHD group (0.8% vs. 0.3%) as shown in Table 2.

**Table 1:** Baseline characteristics of patients with complex CHD and non-complex CHD.

Characteristic	Complex CHD (*n* = 260)	Non-complex CHD (*n* = 2,524)	*P*-value
Male/Female, *n*	143/117	1,074/1,450	<0.001
Age (years)	1.83 (0.67–3.98)	3.10 (1.20–7.17)	<0.001
Height (cm)	82.0 (68.0–98.0)	95.0 (77.0–123.0)	<0.001
Height SDS	−1.28 (−2.31 to −0.12)	−0.33 (−1.42 to 0.65)	<0.001
<−3 SDS, *n* (%)	26 (10.0)	146 (5.8)	0.007
≥−3<−2 SDS, *n* (%)	52 (20.0)	236 (9.3)	<0.001
≥−2<−1 SDS, *n* (%)	69 (26.5)	439 (17.4)	<0.001
≥−1 SDS, *n* (%)	113 (43.5)	1,703 (67.5)	<0.001
Weight (kg)	10.0 (7.5–14.4)	14.0 (10.0–23.0)	<0.001
Weight SDS	−1.15 (−1.83 to −0.43)	−0.47 (−1.25 to 0.42)	<0.001
<−3SDS, *n* (%)	21 (8.1)	111 (4.4)	0.008
≥−3<−2SDS, *n* (%)	50 (19.2)	227 (9.0)	<0.001
≥−2<−1SDS, *n* (%)	82 (31.5)	487 (19.3)	<0.001
≥−1SDS, *n* (%)	107 (41.2)	1,699 (67.3)	<0.001
GR, *n* (%)	92 (35.4)	404 (16.0)	<0.001
BMI (kg/m^2^)	15.14 (13.89–16.62)	15.58 (14.18–17.50)	0.303
Cyanosis, *n* (%)^a^	157 (74.1)	31 (2.1)	<0.001
Wheezing, *n* (%)^b^	7 (3.3)	8 (0.5)	<0.001
Pneumonia, *n* (%)^c^	18 (6.9)	84 (3.3)	0.003
PAH, *n* (%)	45 (17.3)	451 (17.9)	0.822
VSD size (mm)	11.9 (9.6–16.2)	2.1 (0.0–8.2)	<0.001
TP (g/L)	62.2 (57.4–66.1)	64.3 (60.3–68.3)	<0.001
ALB (g/L)	41.4 (38.8–43.7)	42.0 (29.8–44.4)	<0.001
TC (mmol/L)	3.4 (2.9–4.0)	3.8 (3.2–4.3)	<0.001
LDL (mmol/L)	2.0 (1.6–2.4)	2.2 (1.8–2.6)	<0.001
CO2CP (mmol/L)	21.0 (18.3–23.0)	23.0 (21.0–25.0)	<0.001
RBC (10^12^/L)	5.2 (4.5–6.0)	4.4 (4.1–4.7)	<0.001
HGB (g/L)	128.0 (114.0–157.0)	121.0 (110.0–129.0)	<0.001
PT (sec)	11.9 (11.3–13.0)	11.5 (11.0–12.1)	<0.001

Data are presented as median (IQR) for continuous variables and *n* (%) for categorical variables. ALB, albumin; BMI, body mass index; CO_2_CP, carbon dioxide combining power; GR, growth retardation; HGB, hemoglobin; LDL, low-density lipoprotein; PAH, pulmonary arterial hypertension; PT, prothrombin time; RBC, red blood cell counts; SDS, standard deviation score; TC, total cholesterol; TP, total protein; VSD, ventricular septal defect.

a A total of 1,670 data in Cyanosis.

b A total of 1,670 data in Wheezing.

c A total of 1,670 data in Pneumonia.

**Table 2:** Prognostic indicators of patients with complex CHD and non-complex CHD.

Indicators	Complex CHD (*n* = 260)	Non-complex CHD (*n* = 2,524)	*P*-value
LOS (days)	18.00 (14.00–23.00)	13.00 (9.00–16.00)	<0.001
Death, *n* (%)	2 (0.8)	7 (0.3)	0.183

Data are presented as median (IQR) for continuous variables and *n* (%) for categorical variables. LOS, length of stay.

### 3.2 Growth profile of CHD patients

**Figure 2:**
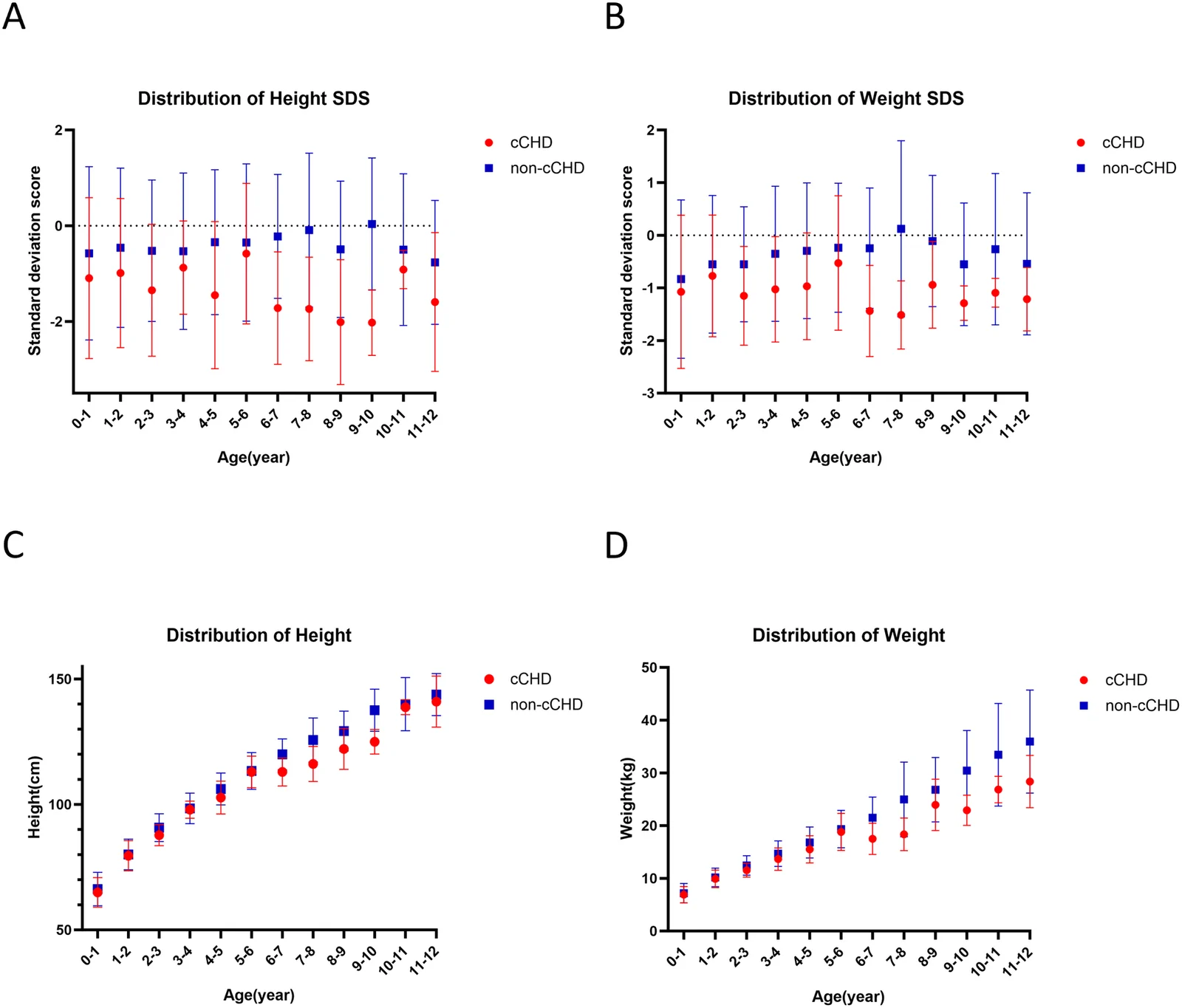
Age-stratified cross-sectional growth profiles according to the type of CHD. **(A)** The distribution of height SDS; **(B)** the distribution of weight SDS; **(C)** the distribution of height; **(D)** the distribution of weight. Each point represents the group mean and the error bars represent standard deviations. cCHD, complex CHD; SDS, standard deviation score.

Analysis of age-stratified cross-sectional growth profiles revealed significant disparities between cardiac structural abnormalities. Patients with complex CHD had lower absolute heights and weights, corresponding to a more severe GR phenotype. When stratified by age, growth deficits (short stature and underweight) were evident in both groups among children under 6 years of age. However, a key divergence was noted in the 6-to-12-year age bracket: while the growth parameters of non-complex CHD group trended towards normalization, the complex CHD group continued to exhibit a significant growth delay. This was confirmed by standardized metrics; both the mean height SDS and weight SDS were significantly lower in the complex CHD group compared to the non-complex group, especially for the 6-to-12-years old patients (Figure 2 and Supplementary Tables S1–S5).

### 3.3 Factors associated with GR in CHD patients

In the training cohort, univariate logistic regression analysis identified several factors significantly associated with GR, including CHD group (complex vs. non-complex), age, LOS, pneumonia, VSD size, PAH, TP, ALB, and HGB (Supplementary Table S6). These variables were subsequently entered into a stepwise multivariate logistic regression model to identify independent factors associated with GR. The final model retained four factors significantly associated with GR, including CHD group (complex), age, presence of PAH, and ALB level (Table 3).

**Table 3:** Factors associated with GR with bivariable and regression analysis.

Variables	Bivariable		Multivariable		VIF
	OR (95% CI)	*P*	OR (95% CI)	*P*	
Group	0.348 (0.264–0.458)	<0.001	0.435 (0.324–0.584)	<0.001	1.017
Age	0.886 (0.861–0.911)	<0.001	0.902 (0.876–0.928)	<0.001	1.039
PAH	1.840 (1.462–2.315)	<0.001	1.595 (1.250–2.036)	<0.001	1.035
ALB	0.931 (0.907–0.955)	<0.001	0.956 (0.930–0.982)	0.001	1.051

ALB, albumin; CI, confidence interval; OR, odds ratio; PAH, pulmonary arterial hypertension; VIF, variance inflation factor. Group refers to the stratification of patients into complex and non-complex groups based on the severity of their CHD.

### 3.4 Construct a nomogram of GR for CHD patients and conduct internal validation

**Figure 3:**
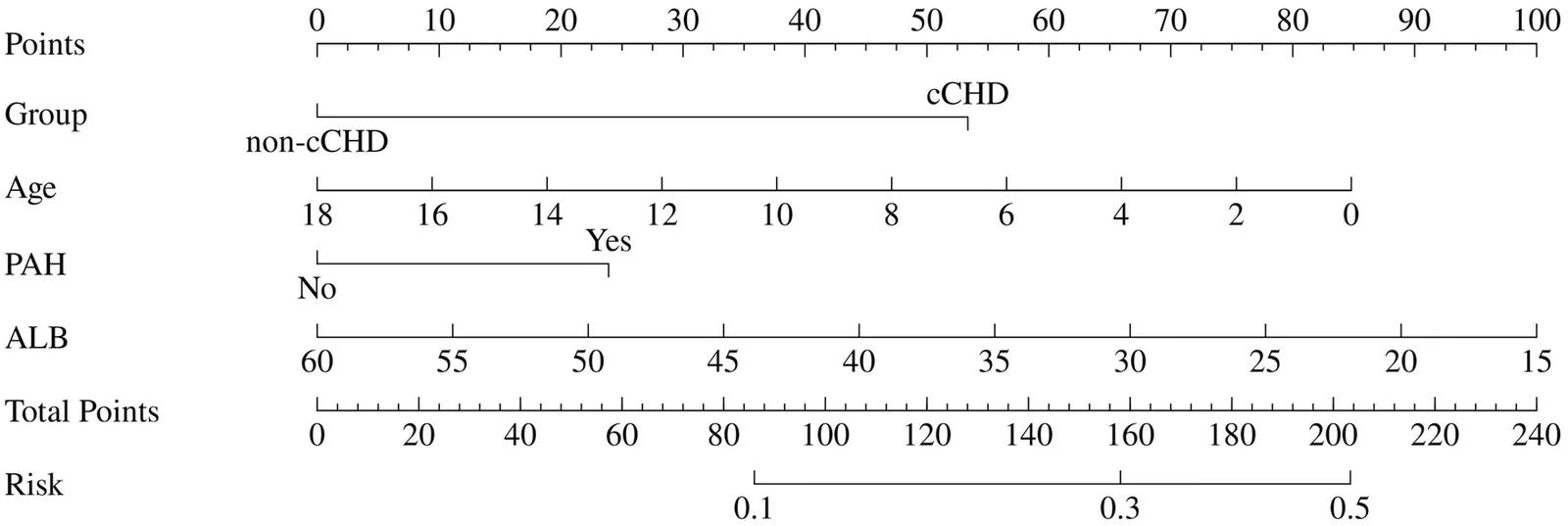
Nomogram for estimating the probability of GR in patients with CHD. To estimate the probability of GR in patients with CHD, the specific values for group, age, PAH, and ALB are located on their respective variable axes. Lines are drawn upward to determine the points assigned to each variable; the sum of these points is located on the Total Points axis, and a line is drawn downward to the Risk axis to determine the probability of GR. ALB, albumin; cCHD, complex CHD; non-cCHD, non-complex CHD; PAH, pulmonary arterial hypertension.

**Figure 4:**
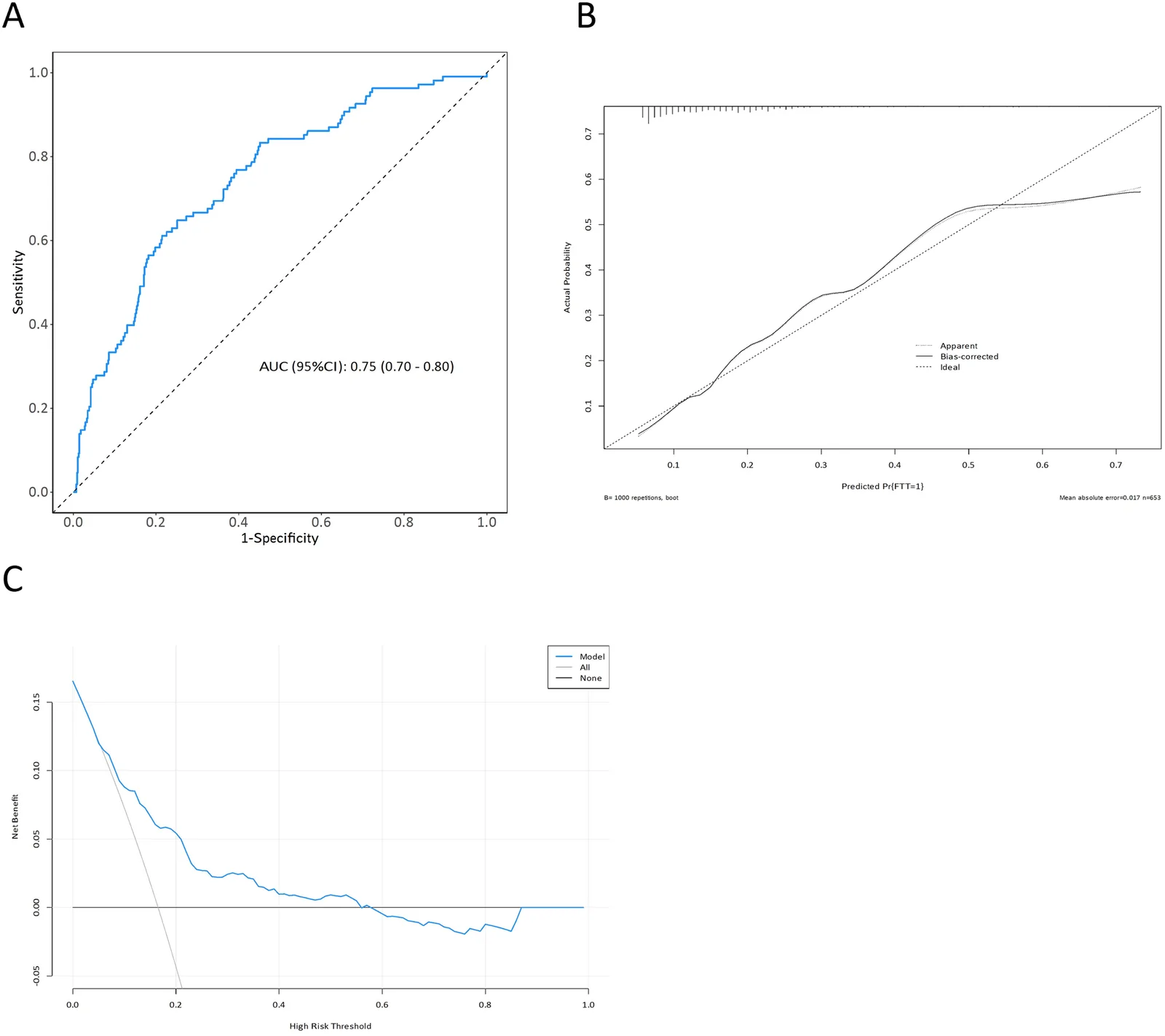
**(A)** The ROC curve of the nomogram in the validation cohort. **(B)** The calibration curve of the nomogram in the validation cohort. **(C)** The DCA curves of the nomogram in the validation cohort.

A nomogram was constructed based on the above four independent factors identified in the multivariate model to estimate the probability of GR in patients with CHD (Figure 3, See Supplementary Figure S1 for detailed interpretation). There were 386 patients with GR in the training cohort (*n* = 2,117) and 110 in the validation cohort (*n* = 667). The performance of the nomogram was evaluated in the temporal internal validation cohort. The model demonstrated discriminative ability with an AUC of 0.75 (95% CI: 0.70–0.80) (Figure 4A). Calibration curves showed agreement between the estimated probabilities and the observed results for both cohorts (Figure 4B). In addition, DCA demonstrated a potential net benefit across a practical probability threshold range of 1%–57%, supporting its potential use in clinical practice (Figure 4C).

## 4 Discussion

GR represents a significant concern in the growth and development of patients with CHD. In this study, we established a 10-year retrospective cohort to investigate the clinical characteristics, growth profiles, and factors associated with GR in CHD. For the first time, we identified complex CHD as an independent factor associated with existing GR, and characterized the growth profiles for both complex and non-complex CHD subtypes. Our findings underscore the necessity of closely monitoring growth and development in the clinical management of CHD patients, particularly those with complex CHD.

CHD is the most prevalent congenital defect globally and ranks first among birth defects in China, posing a substantial threat to child health. Fortunately, advancements in cardiac surgery and interventional therapies have dramatically reduced mortality rates. As patient survival improves, the clinical focus has shifted towards long-term quality of life, making the management of comorbidities like GR increasingly important. Previous studies have reported a high prevalence of GR in CHD patients (22–24). For instance, a large cross-sectional study reported a GR prevalence of 36.9% in Chinese CHD (16), while another Chinese cohort study reported a preoperative GR rate of 23.3% (25). In our cohort, we identified an overall GR prevalence of 17.8% in CHD patients, with rates of short stature and underweight at 16.5% and 14.7%, respectively. Critically, these rates were substantially higher in patients with complex CHD, where the prevalence of short stature reached 30.0%, underweight 27.3%, and GR 35.4%. These findings highlight the disproportionate burden of growth failure in the complex CHD subpopulation and reinforce the need for targeted monitoring.

To provide a comprehensive context for the adverse impact of CHD on growth, we compared our findings with the growth benchmarks of the general pediatric population. According to the Growth Standards for Children under 7 Years of Age and the Chinese National Survey, which serve as the normative references for healthy Chinese children, the theoretical prevalence of growth retardation (defined as height or weight <−2 SDS) in a healthy population is approximately 2.3% (19, 20). In stark contrast, our cohort exhibited a substantially elevated burden of growth impairment. The overall prevalence of GR in our CHD population was 17.8%, indicating a nearly 7.7-fold higher prevalence compared to healthy children. Furthermore, this burden was disproportionately concentrated among patients with complex CHD, where the prevalence escalated to 35.4%. This direct comparison highlights that CHD, particularly complex anatomical variants, imposes a significant and deleterious burden on children's growth and development, far exceeding the baseline levels observed in the general population.

Numerous studies have sought to identify the factors associated with GR in children with CHD. Prior research in China has implicated preoperative anemia, left ventricular systolic dysfunction, younger age, genetic syndrome, and low birth weight were associated factors for GR in CHD patients (16). Another study indicated that cyanosis is also associated factor for GR (26). However, no single study has comprehensively integrated the full spectrum of potential influencing indicators. To address this gap, our study retrospectively analyzed the data of a decade-spanning retrospective cohort, encompassing a wide array of demographic, laboratory, and echocardiographic results. This comprehensive approach was designed to capture as many potential risk factors as possible. Among all variables analyzed, we found complex CHD is a powerful and significant risk factor for GR. Even after adjusting for multiple confounding variables, complex CHD remained an independent factor for impaired growth in CHD patients.

We also highlight the critical importance of monitoring growth in patients with complex CHD. Previous research has primarily focused on the growth patterns of CHD with cyanosis. For instance, an Indonesia cross-sectional study reported a higher prevalence of GR in patients with cyanotic CHD compared to those with acyanotic CHD (27). Another cross-sectional study involving children aged 24–69 months found that the proportions of underweight and stunting were 42.3% and 38.5%, respectively, among children with cyanotic CHD (28). Our study is the first to demonstrate the high prevalence of GR in patients with complex CHD from the perspective of cardiac structural malformations. By stratifying patients based on anatomical severity rather than the presence of cyanosis alone, we offer a more refined understanding of growth impairment risk. We found that patients with complex CHD exhibited substantially higher prevalence of short stature (30.0%), underweight (27.3%), and GR (35.4%). Therefore, early screening and proactive intervention are imperative to mitigate poor growth outcomes in this high-risk group.

To translate our findings into a clinically useful tool, we constructed a nomogram to quantify the factors associated with GR based on four key variables: complex CHD, age, presence of PAH, and ALB levels. The analysis revealed that complex CHD remained an independent factor associated with GR in these patients. Beyond the complexity of the cardiac defect itself, we consider age, presence of PAH, and ALB to be significant contributing factors. The contributions of the other variables are likely mediated by several pathophysiological mechanisms. First, younger age, particularly during infancy, is associated with higher energy and nutritional demands for growth and development. During this critical period, hemodynamic instability caused by CHD can have a more pronounced negative impact on growth (29, 30). Second, PAH may impair linear growth and weight gain by inducing hypoxemia, activating the HIF-1α pathway, triggering chronic inflammation and oxidative stress, and consequently disrupting the growth hormone-insulin-like growth factor-1 axis (31). Finally, low ALB is a direct indicator of malnutrition, which impairs essential tissue synthesis and repair. These findings underscore the critical importance of early and comprehensive monitoring and intervention for patients with complex CHD. The established nomogram model provides a practical tool for the early identification of high-risk patients and facilitates targeted therapeutic strategies.

A comprehensive and integrated management approach is imperative for patients with complex CHD, emphasizing the accurate identification of patients currently presenting with GR to guide clinical management. For the first time, we described the growth characteristics for both complex and non-complex CHD patients. We found that among children aged 0–12 years, those with complex CHD exhibited significant GR in both height and weight compared to their non-complex CHD counterparts. This disparity may be attributed to the higher prevalence of concomitant conditions in complex CHD, such as cyanosis (32), PAH (33, 34), chronic hypoxia (32, 35, 36), genetic abnormalities (37), malnutrition (38–41), and hemodynamic instability (15, 26, 42). Notably, CHD patients presenting at a younger age (0–6 years), regardless of disease complexity, showed pronounced deficits in height and weight, likely due to their more severe clinical manifestations during this critical developmental window. In contrast, non-complex CHD patients who sought medical attention later (6–12 years) demonstrated a trend of catch-up growth in height and weight, possibly because they had passed the most vulnerable period for growth disturbances. Consequently, we strongly recommend that patients with complex CHD receive prompt cardiological evaluation followed by early referral to specialized growth and development clinics. Without timely intervention, GR can exert persistent negative effects throughout the lifespan. Patients may continue to experience slow height velocity and inadequate weight gain, ultimately compromising their final adult height.

There are some limitations in this study. First, as a single-center, retrospective analysis of surgically-managed patients, it may be subject to selection and information bias, and its findings may not be generalizable to patients with milder, non-surgically managed CHD. Second, our assessment of GR relied on static anthropometric parameters rather than longitudinal indicators like growth velocity. Additionally, several important factors known to influence growth, such as birth weight, prematurity, genetic syndromes and socioeconomic status, were not available in our retrospective dataset. Finally, while the nomogram demonstrated acceptable discriminative ability in the temporal internal validation cohort, its generalizability to other populations requires further external validation. To address the limitation regarding the dynamic assessment of growth, we have initiated a prospective follow-up component for this cohort. In our future work, we plan to conduct long-term follow-up visits to collect longitudinal growth data. This will allow us to dynamically analyze the association between the age-stratified cross-sectional growth profiles of CHD patients and GR, thereby further validating the conclusions drawn from this retrospective study. Future research should prioritize prospective, multi-center designs to minimize bias and incorporate a broader spectrum of CHD severity and should explore the application of machine learning algorithms to potentially enhance the identification accuracy and clinical utility of the model.

## 5 Conclusion

This study is the first to demonstrate that complex CHD is an independent risk factor for GR and is associated with a high prevalence of growth impairment. We characterized the growth profiles for both complex and non-complex CHD patients, revealing comprehensive GR across height and weight measurements in the complex CHD group compared to their non-complex counterparts. These findings underscore the critical need for clinicians to incorporate growth monitoring into the routine assessment of CHD patients, with particular emphasis on those with complex conditions. Furthermore, we developed and validated a model for assessing the probability of GR in CHD patients. This model provides clinicians with a simple and intuitive tool for the early identification of children at high risk for GR. Identifying these associated factors allows for early recognition of children at high risk for growth impairment, facilitating timely interventions that may improve their growth trajectories. Future prospective, multi-center studies are warranted to externally validate our model and further enhance its clinical utility.

## Data Availability

The original contributions presented in the study are included in the article/Supplementary Material, further inquiries can be directed to the corresponding authors.
