# Supplementary material for: Impact of anatomic complexity of congenital heart disease on growth retardation: a large retrospective cohort study
Source: Front Cardiovasc Med. 2026 Sep 10;13:1907307. doi: 10.3389/fcvm.2026.1907307 (PMC13601207; doi:10.3389/fcvm.2026.1907307)
Supplement: Supplementary file 1 [file Datasheet1.docx]

Supplementary Material

**Table S1.** The distribution of height SDS according to the different type of CHD and age.

| Age | complex CHD | non-complex CHD |
| --- | --- | --- |
| 0-1 | -1.10±1.68 | -0.58±1.81 |
| 1-2 | -0.99±1.56 | -0.46±1.66 |
| 2-3 | -1.35±1.38 | -0.52±1.48 |
| 3-4 | -0.88±0.98 | -0.53±1.63 |
| 4-5 | -1.45±1.54 | -0.34±1.52 |
| 5-6 | -0.58±1.47 | -0.35±1.64 |
| 6-7 | -1.72±1.18 | -0.23±1.29 |
| 7-8 | -1.74±1.08 | -0.09±1.61 |
| 8-9 | -2.01±1.30 | -0.49±1.43 |
| 9-10 | -2.02±0.68 | 0.04±1.38 |
| 10-11 | -0.93±0.40 | -0.50±1.58 |
| 11-12 | -1.60±1.45 | -0.77±1.29 |

Data are presented as mean ± SDS.

**Table S2.** The distribution of weight SDS according to the different type of CHD and age.

| Age | complex CHD | non-complex CHD |
| --- | --- | --- |
| 0-1 | -1.08±1.45 | -0.83±1.51 |
| 1-2 | -0.77±1.15 | -0.55±1.31 |
| 2-3 | -1.15±0.94 | -0.55±1.09 |
| 3-4 | -1.03±1.00 | -0.35±1.28 |
| 4-5 | -0.97±1.01 | -0.29±1.29 |
| 5-6 | -0.53±1.28 | -0.24±1.22 |
| 6-7 | -1.44±0.87 | -0.25±1.14 |
| 7-8 | -1.51±0.65 | 0.12±1.68 |
| 8-9 | -0.94±0.82 | -0.11±1.25 |
| 9-10 | -1.29±0.33 | -0.55±1.17 |
| 10-11 | -1.09±0.27 | -0.26±1.44 |
| 11-12 | -1.21±0.60 | -0.54±1.35 |

Data are presented as mean ± SDS.

**Table S3.** The distribution of height according to the different type of CHD and age.

| Age | complex CHD | non-complex CHD |
| --- | --- | --- |
| 0-1 | 64.94±5.92 | 66.31±6.66 |
| 1-2 | 79.55±6.07 | 80.10±6.15 |
| 2-3 | 87.76±4.20 | 90.78±5.55 |
| 3-4 | 97.93±3.41 | 98.44±6.13 |
| 4-5 | 102.73±6.59 | 106.18±6.37 |
| 5-6 | 113.00±6.32 | 113.36±7.31 |
| 6-7 | 113.00±5.62 | 119.98±6.21 |
| 7-8 | 116.14±7.01 | 125.70±8.77 |
| 8-9 | 122.14±8.13 | 129.22±7.98 |
| 9-10 | 125.00±4.90 | 137.55±8.40 |
| 10-11 | 138.80±3.03 | 139.98±10.61 |
| 11-12 | 141.00±10.18 | 143.82±8.44 |

Data are presented as mean ± SDS.

**Table S4.** The distribution of weight according to the different type of CHD and age.

| Age | complex CHD | non-complex CHD |
| --- | --- | --- |
| 0-1 | 6.91±1.54 | 7.17±1.85 |
| 1-2 | 9.91±1.67 | 10.17±1.76 |
| 2-3 | 11.53±1.32 | 12.44±1.84 |
| 3-4 | 13.67±2.31 | 14.68±2.43 |
| 4-5 | 15.50±2.56 | 16.79±2.94 |
| 5-6 | 18.81±3.52 | 19.34±3.55 |
| 6-7 | 17.50±2.97 | 21.49±3.94 |
| 7-8 | 18.36±2.97 | 24.97±7.07 |
| 8-9 | 23.93±4.89 | 26.81±6.10 |
| 9-10 | 22.90±2.88 | 30.43±7.62 |
| 10-11 | 26.84±2.53 | 33.43±9.74 |
| 11-12 | 28.36±4.96 | 35.95±9.79 |

Data are presented as mean ± SDS.

**Table S5.** The distribution of patients across different age groups by CHD complexity.

| Age (years) | complex CHD (n) | non-complex CHD (n) |
| --- | --- | --- |
| 0-1 | 96 | 541 |
| 1-2 | 40 | 375 |
| 2-3 | 46 | 327 |
| 3-4 | 15 | 255 |
| 4-5 | 11 | 165 |
| 5-6 | 8 | 121 |
| 6-7 | 6 | 105 |
| 7-8 | 7 | 76 |
| 8-9 | 7 | 58 |
| 9-10 | 5 | 72 |
| 10-11 | 5 | 62 |
| 11-12 | 7 | 41 |

**Table S6.** Univariate logistic regression analysis of factors associated with GR.

| Variables | Model 1 | | Model 2 | | Model 3 | |
| --- | --- | --- | --- | --- | --- | --- |
|  | OR (95%CI) | *P* | OR (95%CI) | *P* | OR (95%CI) | *P* |
| Group | 0.348 (0.264–0.458) | <0.001 | 0.441 (0.313–0.621) | <0.001 | 0.435 (0.324–0.584) | <0.001 |
| Age | 0.886 (0.861–0.911) | <0.001 | 0.904 (0.877–0.932) | <0.001 | 0.902 (0.876–0.928) | <0.001 |
| PAH | 1.840 (1.462–2.315) | <0.001 | 1.564 (1.222–2.002) | <0.001 | 1.595 (1.250–2.036) | <0.001 |
| ALB | 0.931 (0.907–0.955) | <0.001 | 0.956 (0.930–0.982) | 0.001 | 0.956 (0.930–0.982) | 0.001 |
| VSD size | 1.052 (1.035–1.070) | <0.001 | 1.010 (0.990–1.031) | 0.325 | - | - |
| HGB | 0.993 (0.988–0.999) | 0.025 | 0.998 (0.992–1.004) | 0.587 |  |  |
| LOS | 1.036 (1.024–1.048） | <0.001 | - | - | - | - |
| Pneumonia | 2.161 (1.277–3.656) | 0.004 | - | - | - | - |
| TP | 0.946 (0.932–0.961) | <0.001 | - | - | - | - |

Abbreviations: ALB, albumin; CI, confidence interval; HGB, hemoglobin; LOS, length of stay; OR, odds ratio; PAH, pulmonary arterial hypertension; TP, total protein; VSD, ventricular septal defect. Group refers to the stratification of patients into complex and non-complex groups based on the severity of their CHD.

**Figure S1** Nomogram to visually predict the occurrence of GR in patients with CHD. Each risk factor is assigned a specific score along the vertical axis according to its value. For example, a patient with complex CHD who is 4 years of age, presents with PAH, and has an ALB level of 35 g/L, would obtain a total score of 199, corresponding to a probability of GR occurrence of approximately 0.5.
